# Effect of eicosapentaenoic acid/docosahexaenoic acid on coronary high-intensity plaques detected with non-contrast T1-weighted imaging (the AQUAMARINE EPA/DHA study): study protocol for a randomized controlled trial

**DOI:** 10.1186/s13063-017-2353-1

**Published:** 2018-01-08

**Authors:** Kazuhiro Nakao, Teruo Noguchi, Yasuhide Asaumi, Yoshiaki Morita, Tomoaki Kanaya, Masashi Fujino, Hayato Hosoda, Shuichi Yoneda, Shoji Kawakami, Toshiyuki Nagai, Kensaku Nishihira, Takahiro Nakashima, Reon Kumasaka, Tetsuo Arakawa, Fumiyuki Otsuka, Michio Nakanishi, Yu Kataoka, Yoshio Tahara, Yoichi Goto, Haruko Yamamoto, Toshimitsu Hamasaki, Satoshi Yasuda

**Affiliations:** 1 Department of Cardiovascular Medicine, National Cerebral and Cardiovascular Center, 5-7-1 Fujishiro-dai, Suita, Osaka 565-8565 Japan; 2 Department of Radiology, National Cerebral and Cardiovascular Center, 5-7-1 Fujishiro-dai, Suita, Osaka 565-8565 Japan; 3 Center for Advancing Clinical and Translational Science, National Cerebral and Cardiovascular Center, 5-7-1 Fujishiro-dai, Suita, Osaka 565-8565 Japan; 4 Department of Data Sceince, National Cerebral and Cardiovascular Center, 5-7-1 Fujishiro-dai, Suita, Osaka 565-8565 Japan

**Keywords:** Long-chain n-3 polyunsaturated fatty acids, Eicosapentaenoic acid, Docosahexaenoic acid, High-risk plaque, Cardiac magnetic resonance, Residual risk

## Abstract

**Background:**

Despite the success of HMG-CoA reductase inhibitor (statin) therapy in reducing atherosclerotic cardiovascular events, a residual risk for cardiovascular events in patients with coronary artery disease (CAD) remains. Long-chain n-3 polyunsaturated fatty acids (LC n-3 PUFAs), especially eicosapentaenoic acid (EPA) and docosahexaenoic acid (DHA), are promising anti-atherosclerosis agents that might reduce the residual CAD risk. Non-contrast T1-weighted imaging (T1WI) with cardiac magnetic resonance (CMR) less invasively identifies high-risk coronary plaques as high-intensity signals. These high-intensity plaques (HIPs) are quantitatively assessed using the plaque-to-myocardium signal intensity ratio (PMR). Our goal is to assess the effect of EPA/DHA on coronary HIPs detected with T1WI in patients with CAD on statin treatment.

**Methods/design:**

This prospective, controlled, randomized, open-label study examines the effect of 12 months of EPA/DHA therapy and statin treatment on PMR of HIPs detected with CMR and computed tomography angiography (CTA) in patients with CAD. The primary endpoint is the change in PMR after EPA/DHA treatment. Secondary endpoints include changes in Hounsfield units, plaque volume, vessel area, and plaque area measured using CTA. Subjects are randomly assigned to either of three groups: the 2 g/day EPA/DHA group, the 4 g/day EPA/DHA group, or the no-treatment group.

**Discussion:**

This trial will help assess whether EPA/DHA has an anti-atherosclerotic effect using PMR of HIPs detected by CMR. The trial outcomes will provide novel insights into the effect of EPA/DHA on high-risk coronary plaques and may provide new strategies for lowering the residual risk in patients with CAD on statin therapy.

**Trial registration:**

The University Hospital Medical Information Network (UMIN) Clinical Trials Registry, ID: UMIN000015316. Registered on 2 October 2014.

**Electronic supplementary material:**

The online version of this article (doi:10.1186/s13063-017-2353-1) contains supplementary material, which is available to authorized users.

## Background

Evidence strongly suggests that lowering low-density lipoprotein (LDL)-cholesterol levels with statins reduces the risk of cardiovascular disease as primary and secondary prevention of atherosclerotic cardiovascular events [1–7]. However, many clinical trials have found a significant residual risk for cardiovascular events even in the setting of optimal LDL-cholesterol reduction with statins [8, 9]. Thus, there is a need to establish strategies beyond lowering LDL-cholesterol levels with statins to reduce the risk of cardiovascular events.

Epidemiologic studies have shown that increasing the intake of long-chain n-3 polyunsaturated fatty acids (n-3 PUFAs), especially eicosapentaenoic acid (EPA) and docosahexaenoic acid (DHA), is inversely associated with cardiovascular or cerebrovascular disease incidence [10–15]. The LC n-3 PUFAs have a different anti-atherosclerotic mechanism from statins, including triglyceride and remnant lipoprotein-lowering effects [16]. In experimental study, EPA/DHA treatments significantly attenuated the development and destabilization of atherosclerotic plaques [17]. Although meta-analysis failed to demonstrate the inhibitory effect of cardiovascular events by LC n-3 PUFA supplementation, the effects of high-dose LC n-3 PUFAs on cardiovascular outcome should be discussed (most previous and on-going studies use 1 g EPA/DHA) [18]. Indeed, higher-dose EPA (1.8 g) could reduce the residual risk of cardiovascular diseases in patients receiving LDL lowering with statins [19]. And previous human studies have investigated the effect of high-dose EPA on plaque stability or plaque composition [20, 21]. Therefore, the present study focuses on the effects of high-dose administration of EPA/DHA on coronary plaques.

We have reported that coronary high-intensity plaques (HIPs) detected with non-contrast T1-weighted imaging (T1WI) on cardiac magnetic resonance (CMR) are associated with computed tomography angiography (CTA) – and intravascular ultrasound-derived – features of high-risk plaques [22, 23]. HIPs, which can be uniquely assessed using the plaque-to-myocardium signal intensity ratio (PMR), [22] are significantly associated with future coronary events [24]. More recently, in the AQUAMARINE (Attempts at Plaque Vulnerability Quantification with Magnetic Resonance Imaging Using Non-contrast T1-weighted Technic) study, we have demonstrated that intensive lipid-lowering therapy with pitavastatin reduces the PMR of coronary HIPs by approximately 25% [25]. Thus, non-contrast T1WI is now being established as a less-invasive technique for evaluating high-risk coronary plaques, and as such, might be a promising predictive factor in patients at high risk.

The present AQUAMARINE-EPA/DHA study is designed to assess the anti-atherogenic effect of EPA/DHA in an exploratory manner. We will study the change in PMR of coronary HIPs detected using CMR after 12 months of EPA/DHA therapy.

## Methods/design

### Aim

The aim of this study is to assess the effect of EPA/DHA on PMR of HIPs in patients with coronary artery disease (CAD) detected using non-contrast T1WI imaging with CMR.

### Study design

**Fig. 1:**
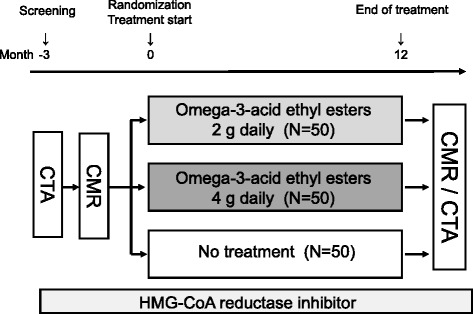
Study flow of this study

This is a single-center, triple-arm, parallel-group, randomized controlled, open-label, superiority trial examining the effect of 12 months of EPA/DHA therapy on coronary HIPs in patients with CAD between May 2014 and April 2018. Eligible subjects are randomly assigned to the 2 g/day EPA/DHA group, the 4 g/day EPA/DHA group, or the no-treatment group (allocation rate 1:1:1). The randomization stratified on following variables (age, gender, presence or absence of type 2 diabetes mellitus, and PMR of the primary coronary lesion measured with non-contrast T1WI) (Fig. 1).

### Patient eligibility and recruitment

Patients aged 20 years or older with proven or suspected CAD receiving statin therapy are eligible for the study. It is necessary for eligible subjects to achieve an optimal LDL-cholesterol level (LDL-cholesterol < 100 mg/dl) recommended by the Japan Atherosclerosis Society. Patients are excluded if they are scheduled for percutaneous coronary intervention (PCI) or coronary artery bypass grafting (CABG) or had taken EPA/DHA within 12 weeks before providing informed consent, type 1 diabetes mellitus or type 2 diabetes mellitus with uncontrolled hyperglycemia (glycosylated hemoglobin (HbA1C) ≥ 8.0%), or the presence of bleeding (e.g., hemophilia, capillary fragility, gastrointestinal tract ulceration, urinary tract bleeding, hemoptysis, vitreous hemorrhage).

### Study procedures

**Fig. 2:**
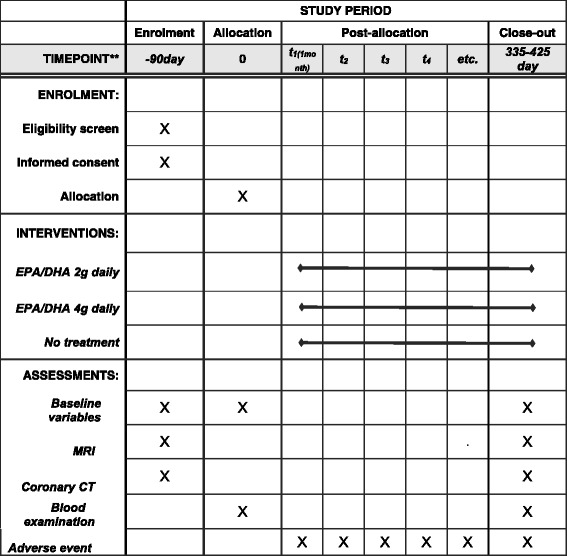
Example template of recommended content for the schedule of enrollment, interventions, and assessments

Eligible patients are randomly assigned to the 2 g/day EPA/DHA group, the 4 g/day EPA/DHA group, or the no-treatment group. Prospective, serial, non-contrast T1WI and CTA examinations are performed at baseline and after 12 months of EPA/DHA treatment (Fig. 2).

#### Inclusion and exclusion criteria

##### Inclusion criteria

Subjects must meet all of the following criteria:

1. Be aged 20 years or older
2. Diagnosed with dyslipidemia and have received instructions for a 3–6-month lifestyle modification
3. Diagnosed with coronary artery disease with > 25% coronary stenosis measured by CTA or invasive coronary angiography (CAG)
4. On statin therapy at a fixed dose and administration method for at least 4 weeks before providing informed consent
5. Have at least one coronary plaque with PMR ≥ 1.0 with non-contrast T1WI on the screening examination
6. Serum LDL-cholesterol level < 100 mg/dL on the screening test
7. Capable of visiting the study centers on an outpatient basis
8. Capable of providing written consent before study participation

##### Exclusion criteria

Those who meet any of the following criteria are ineligible for the study:

1. Use of LC n-3 PUFA preparations within 12 weeks before providing informed consent
2. Scheduled to undergo PCI or CABG during the observation period
3. Presence of type 1 diabetes mellitus or type 2 diabetes mellitus, with uncontrolled hyperglycemia (HbA1C ≥ 8.0%)
4. Clinically apparent impairment of renal function (estimated glomerular filtration rate < 40 ml/min)
5. Presence of bleeding (e.g., hemophilia, capillary fragility, gastrointestinal tract ulceration, urinary tract bleeding, hemoptysis, vitreous hemorrhage)
6. History of hypersensitivity to any component of the investigational product
7. Pregnancy, possible pregnancy, or nursing

### Outcomes

#### Primary endpoint

The primary endpoint is the change in PMR of the primary coronary lesion measured by non-contrast, T1-weighted cardiac imaging from baseline to study completion or discontinuation (Table 1).

**Table 1:** Summary of the study outcome and measures

Primary endpoint
Change in PMR in the primary lesion measured by CMR imaging
Secondary endpoint
Change in PMR in all lesions measured by CMR imaging Percentage change in PMR in the primary lesion measured by CMR imaging Percentage change in PMR in all lesion measured by CMR imaging Changes in Hounsfield units, plaque volume, vessel area, and plaque area in the primary lesion measured by CTA Changes in Hounsfield units, plaque volume, vessel area, and plaque area in all lesions measured by CTA Percentage changes in Hounsfield units, plaque volume, vessel area, and plaque area in the primary lesion measured by CTA Percentage changes in Hounsfield units, plaque volume, vessel area, and plaque area in all lesions measured by CTA
Other endpoint
Changes and percentage changes in triglycerides, total cholesterol, LDL-cholesterol, HDL-cholesterol, non-HDL-cholesterol, VLDL-cholesterol, remnant-like cholesterol, LPL activity, and high-sensitivity C-reactive protein Changes and percentage changes in small, dense LDL and mean LDL particle diameter Changes and percentage changes in EPA, DHA, AA, EPA/AA ratio, and omega-3 index (weight percentage of eicosapentaenoic acid [EPA, C20:5ω3] and docosahexaenoic acid [DHA, C20:6ω3] in total fatty acids out of all lipids) Changes and percentage changes in leptin and adiponectin

*AA* arachidonic acid, *CMR* cardiac magnetic resonance, *CTA* computed tomography angiography, *HDL* high-density lipoprotein, *LDL* low-density lipoprotein, *LPL* lipoprotein lipase, *PMR* plaque-to-myocardium signal intensity ratio, *VLDL* very-low density lipoprotein

#### Secondary endpoint

1. Change in PMR in all lesions measured by non-contrast, T1-weighted CMR imaging from baseline to study completion or discontinuation
2. Percentage change in PMR of the primary lesion measured by non-contrast, T1-weighted CMR imaging from baseline to study completion or discontinuation
3. Percentage change in PMR of all lesions measured by non-contrast, T1-weighted CMR imaging from baseline to study completion or discontinuation
4. Change and percentage change in Hounsfield units, plaque volume, vessel area, and plaque area of the primary lesion measured by CTA from baseline to study completion or discontinuation
5. Change and percentage change in Hounsfield units, plaque volume, vessel area, and plaque area of all lesions measured by CTA from baseline to study completion or discontinuation

#### Other variables

1. Change and percentage change in triglycerides, total cholesterol, LDL-cholesterol, high-density lipoprotein (HDL)-cholesterol, non-HDL-cholesterol, very-low-density lipoprotein (VLDL)-cholesterol, remnant-like lipoprotein cholesterol, lipoprotein lipase (LPL) activity, and high-sensitivity C-reactive protein from baseline to study completion or discontinuation
2. Change and percentage change in small, dense LDL and mean LDL particle diameter from baseline to study completion or discontinuation (LDL diameter is measured using Lipoprint^TM^ system (Quantimetrix))
3. Change and percentage change in EPA, DHA, arachidonic acid (AA), EPA/AA ratio, and LC n-3 PUFA (percentage of EPA [C20:5ω3] and DHA [C22:6ω3] by weight in all fatty acids out of all lipids) from baseline to study completion or discontinuation
4. Change and percentage change in leptin and adiponectin from baseline to study completion or discontinuation

#### Sample size

Based on the result from our previous study, a clinically meaningful change from baseline in PMR was assumed to be − 0.15 in both the 2 g/day and the 4 g/day LC n-3 PUFA groups, and no change in the no-treatment group, with a common standard deviation of 0.2 among the groups. The sample size per group of 46 archives 90% power to detect the assumed difference in changes, using a two-group, one-sided *t* test of 1.25% significance level, where the overall significance level of the one-sided test for the two comparisons (i.e., the 2 g/day group versus the no-treatment group, and the 4 g/day LC n-3 PUFA group versus the no-treatment group) was 2.5% and the Bonferroni adjustment was applied to control the Type I error rate. Allowing for adjustment of withdrawals, missing data or loss to follow-up, 50 subjects per group will be recruited into the trial.

#### Randomization

In this study, dynamic allocation is used in order to ensure an even allocation of factors that may influence the evaluation of the efficacy of anti-hypertensive medications. Dynamic allocation will be performed at the registration/allocation center at the start of therapy, with respect to age, gender, and presence or absence of type II diabetes. The allocation table and the most recent breakdown of allocated subjects in each group will be used for dynamic allocation at the registration/allocation center. However, this allocation table and the information regarding the number of allocated subjects in each group will not be disclosed to the study representative, principal investigator, or investigator until the completion of the study.

The SAS dynamic allocation system will be used for subject registration and allocation. In this system, web-based e-mails and the SAS server owned by the National Cerebral and Cardiovascular Center are used to automatically register and allocate subjects on a 24-h basis without any human operators.

The principal investigator or investigator will obtain consent for participation in the study from the subjects who they consider will have no problems in participation. After confirming that a subject meets all the eligibility criteria, the principal investigator or investigator will enter the necessary items in the portable document format (PDF) version of the registration form and send it to the SAS dynamic allocation system by e-mail for registration. The SAS dynamic allocation system will check the received registration form to see whether or not the eligibility criteria are met and whether or not the subject is already registered. If there are no problems, the system will allocate the subject using the randomized minimization method based on allocation adjustment factors. The results of the allocation will be automatically sent to the e-mail address used to send the registration form. The principal investigator or investigator will start the treatment based on the notification and record it on the Case Report Form (CRF) for each subject.

#### Allocation factors

Dynamic allocation is performed with respect to the following factors:

- Age at the time of consent: younger than 65 years/65 years or older
- Gender: male/female
- Concomitant type II diabetes: present/absent
- Plaque to myocardium ratio (PMR): below 1.35/1.35 or higher in non-contrast T1-weighted CMR images of the main lesion (a cut-off point of PMR that separates high risk from intermediate risk) [24, 26]

#### Blinding

All imaging and laboratory data will be analyzed by an independent attending physician and a radiologist at the National Cerebral and Cardiovascular Center (Japan), including CMR and CTA measurements. These evaluators will be blinded to patient treatment status.

#### Data quality control and management

The principal investigator or investigator must complete CRFs for each subject who provides written informed consent. The study representative or a person designated by the study representative will authorize access to the electronic CRF system for the principal investigator or investigator. CRFs will be prepared in Japanese. Data will be directly entered into the electronic CRF system during CRF preparation. The principal investigator or investigator will confirm that CRFs are accurately and completely prepared and register the data in the electronic CRF system and other systems. The principal investigator will take full responsibility for the accuracy and reliability of all the data entered in the CRFs.

The completed CRFs are the property of the director of the study center. The principal investigator and other investigators must not disclose the information contained in the CRFs to third parties except for regulatory agencies without written approval from the director of the study center. Only investigators can access data.

#### Statistical methods

All the analysis will be made on an intent-to-treat (ITT) basis. The 2 g/day and the 4 g/day EPA/DHA groups will be compared against the no-treatment group for all primary analysis. For the primary outcome, treatment effects between the groups and their 95% confidence intervals are calculated from analysis of covariance (ANCOVA) models with adjustment for baseline values of the outcome measure. To confirm the conclusion from ITT-based analysis, the same analysis is repeated on a per-protocol set (PPS), where criteria for determining the PPS assignment would be established and confirmed by the Steering Committee. For secondary and other outcomes, we will use the chi-squared test for binary outcomes, and a *t* test for continuous outcomes. For subgroup analyses, we will use regression methods with appropriate interaction terms (respective subgroup × treatment group). Multivariable analyses will be based on logistic regression for binary outcomes and linear regression for continuous outcomes. We will examine the residual to assess model assumptions and goodness-of-fit.

All *p* values will be reported to four decimal places with *p* values less than 0.001 reported as *p* < 0.001. Up-to-date versions of SAS (SAS Institute, Cary, NC, USA) will be used to conduct analyses. For all tests, we will use two-sided *p* values with an alpha ≤ 0.05 level of significance

### Study organization

The research group consists of researchers and statisticians at the National Cerebral and Cardiovascular Center, Suita, Japan and an independent data monitoring committee.

#### Monitoring

Adverse events (AE) will be collected during the study periods. At each study visit, the principal investigator or investigator will interview the subject about whether any subjective symptoms have occurred. When an AE occurs, the principal investigator or investigator must promptly take appropriate measures and follow up all subjects experiencing an AE until the symptoms resolve, or any clinically significant abnormal laboratory values have returned to the baseline values. All AEs will be documented in the CRF. The Data Monitoring Committee is independent of the investigators. No interim analysis is planned.

#### Definition of AEs

An AE is defined as any untoward medical occurrence in a subject administered a drug; it does not necessarily have to have a causal relationship with this treatment. An AE can, therefore, be any unfavorable or unintended sign (including a clinically significant laboratory abnormality), symptom, or disease temporally associated with the use of a drug whether or not it is considered related to the drug.

#### Reporting of AEs

When an AE occurs, the principal investigator or investigator must promptly take appropriate measures and follow up all subjects experiencing an AE until the symptoms resolve, or any clinically significant abnormal laboratory values have returned to baseline values, the addition to, or switching of, the investigational product, or there is a satisfactory explanation for the change (for AEs that are permanent or irreversible), regardless of whether the causal relationship of the AE with the investigational product. All AEs will be documented in the CRF. The following information will be documented for each AE: event term; dates of onset and disappearance; frequency; severity; causal relationship between the event and the investigational product (not related or related); action taken for the investigational product; outcome of event; and seriousness. For AEs that are considered to be unrelated to the investigational product, the reason for ruling out the causality should be documented in the comment column of the CRF.

## Discussion

Although the results of many randomized controlled trials using statins have shown the usefulness of LDL-cholesterol-lowering therapy, the extent of coronary artery disease suppression did not exceed approximately 30% [27]. However, optimal management of residual risk following statin therapy is now emerging.

The LC n-3 PUFAs, especially EPA and DHA, exert pleiotropic cardiometabolic effects with a diverse range of actions. The LC n-3 PUFAs can ultimately reduce blood pressure, reduce platelet aggregation, and improve arterial and endothelial function. Importantly, LC n-3 PUFAs do not have a major effect on fasting total cholesterol and LDL-cholesterol levels; they effectively reduces triglyceride and remnant lipoprotein in hyperlipidemic patients [28]. Those effects of LC n-3 PUFAs are beneficial for the cardiovascular system. The present AQUAMARINE-EPA/DHA study is expected to clarify the effect of LC n-3 PUFAs on high-risk coronary plaques.

In the AQUAMARINE pilot study, we found that statin treatment reduces the PMR of coronary HIPs and that the reduction of PMR reflects improvement of high-risk features of HIPs under statin treatment. However, in many plaques, high PMR values remain (PMR > 1.0) even after statin treatment [25]. These results suggest that PMR measured by CMR can be useful not only to evaluate high-risk coronary plaques but also to assess coronary residual risks after statin treatment.

Therefore, this trial will provide novel insights into the effect of EPA/DHA therapy on residual risk in patients with CAD who are taking appropriate statin therapy.

### Trial status

Period of patient recruitment: recruitment started in August 2015.

## Additional file

Additional file 1: SPIRIT 2013 Checklist: recommended items to address in a clinical trial protocol and related documents. (DOC 119 kb)

## Abbreviations

AA: Arachidonic acid
CAD: Coronary artery disease
CMR: Cardiac magnetic resonance
CRF: Case Report Form
CTA: Computed tomography angiography
DHA: Docosahexaenoic acid
EPA: Eicosapentaenoic acid
HDL: High-density lipoprotein
HIP: High-intensity plaque
LC n-3 PUFA: Long-chain n-3 polyunsaturated fatty acid
LDL: Low-density lipoprotein
LPL: Lipoprotein lipase
PCI: Percutaneous coronary intervention
PMR: Plaque-to-myocardium signal intensity ratio
T1WI: T1-weighted imaging
VLDL: Very-low-density lipoprotein
